# Using hierarchical unsupervised learning to integrate and reduce multi-level and multi-paraspinal muscle MRI data in relation to low back pain

**DOI:** 10.1007/s00586-022-07169-z

**Published:** 2022-03-25

**Authors:** Abel Torres-Espin, Anastasia Keller, Gabriel T. A. Johnson, Aaron J. Fields, Roland Krug, Adam R. Ferguson, Alan R. Hargens, Conor W. O’Neill, Jeffrey C. Lotz, Jeannie F. Bailey

**Affiliations:** 1 Department of Neurological Surgery, Brain and Spinal Injury Center (BASIC), Weill Institute for Neuroscience, UC San Francisco, 1001 Potrero Ave, San Francisco, CA 94110 USA; 2 Department of Orthopaedic Surgery, UC San Francisco, 95 Kirkham St, San Francisco, CA 94122 USA; 3 Department of Radiology, UC San Francisco, San Francisco, CA USA; 4 San Francisco Veterans Affairs Medical Center, San Francisco, CA USA; 5 Department of Orthopaedic Surgery, UC San Diego, La Jolla, CA USA

**Keywords:** Lumbar spine, Paraspinal muscles, MRI, Low back pain, Integrative analysis, Multiple factor analysis, Hierarchical unsupervised learning

## Abstract

**Purpose:**

The paraspinal muscles (PSM) are a key feature potentially related to low back pain (LBP), and their structure and composition can be quantified using MRI. Most commonly, quantifying PSM measures across individual muscles and individual spinal levels renders numerous separate metrics that are analyzed in isolation. However, comprehensive multivariate approaches would be more appropriate for analyzing the PSM within an individual. To establish and test these methods, we hypothesized that multivariate summaries of PSM MRI measures would associate with the presence of LBP symptoms (i.e., pain intensity).

**Methods:**

We applied hierarchical multiple factor analysis (hMFA), an unsupervised integrative method, to clinical PSM MRI data from unique cohort datasets including a longitudinal cohort of astronauts with pre- and post-spaceflight data and a cohort of chronic LBP subjects and asymptomatic controls. Three specific use cases were investigated: (1) predicting longitudinal changes in pain using combinations of baseline PSM measures; (2) integrating baseline and post-spaceflight MRI to assess longitudinal change in PSM and how it relates to pain; and (3) integrating PSM quality and adjacent spinal pathology between LBP patients and controls.

**Results:**

Overall, we found distinct complex relationships with pain intensity between particular muscles and spinal levels. Subjects with high asymmetry between left and right lean muscle composition and differences between spinal segments PSM quality and structure are more likely to increase in pain reported outcome after prolonged time in microgravity. Moreover, changes in PSM quality and structure between pre and post-spaceflight relate to increase in pain after prolonged microgravity. Finally, we show how unsupervised hMFA recapitulates previous research on the association of CEP damage and LBP diagnostic.

**Conclusion:**

Our analysis considers the spine as a multi-segmental unit as opposed to a series of discrete and isolated spine segments. Integrative and multivariate approaches can be used to distill large and complex imaging datasets thereby improving the clinical utility of MRI-based biomarkers, and providing metrics for further analytical goals, including phenotyping.

**Supplementary Information:**

The online version contains supplementary material available at 10.1007/s00586-022-07169-z.

## Introduction

Magnetic resonance imaging (MRI) is the imaging modality of choice to depict spinal structural abnormalities that may cause chronic low back pain (LBP). Thanks to high soft tissue contrast, MRI allows assessment of multiple structures including intervertebral disks, nerve roots, contents of the central spinal canal, ligaments, facet joints [1–4], and muscle [5]. While MRI is superb at identifying tissue pathology the clinical relevance of these findings is often uncertain. Only in about 10% of patients can low back pain be attributed to a specific pathology (e.g., vertebral fracture or disk herniation with nerve compression) [6, 7]. The remaining patients are assigned a diagnosis of non-specific LBP (i.e., LBP without a distinct patho-anatomical cause). Disorders of the paraspinal muscles (PSM) are a potential cause of non-specific LBP, although evidence is conflicting [5]. One challenge in defining the relationship between PSM pathology and LBP is the complicated relationships between individual muscles, between muscles and spinal segments, and between spinal segments and regional lumbar biomechanics. Prior work has relied on summary metrics from a particular muscle and/or spinal level, which might not be sufficient to determine the relationship between PSM’s and LBP. Analytic methods that characterize multiple muscle and multiple spinal-level relationships may lead to a clearer understanding of the role of PSM’s in LBP.

**Fig. 1:**
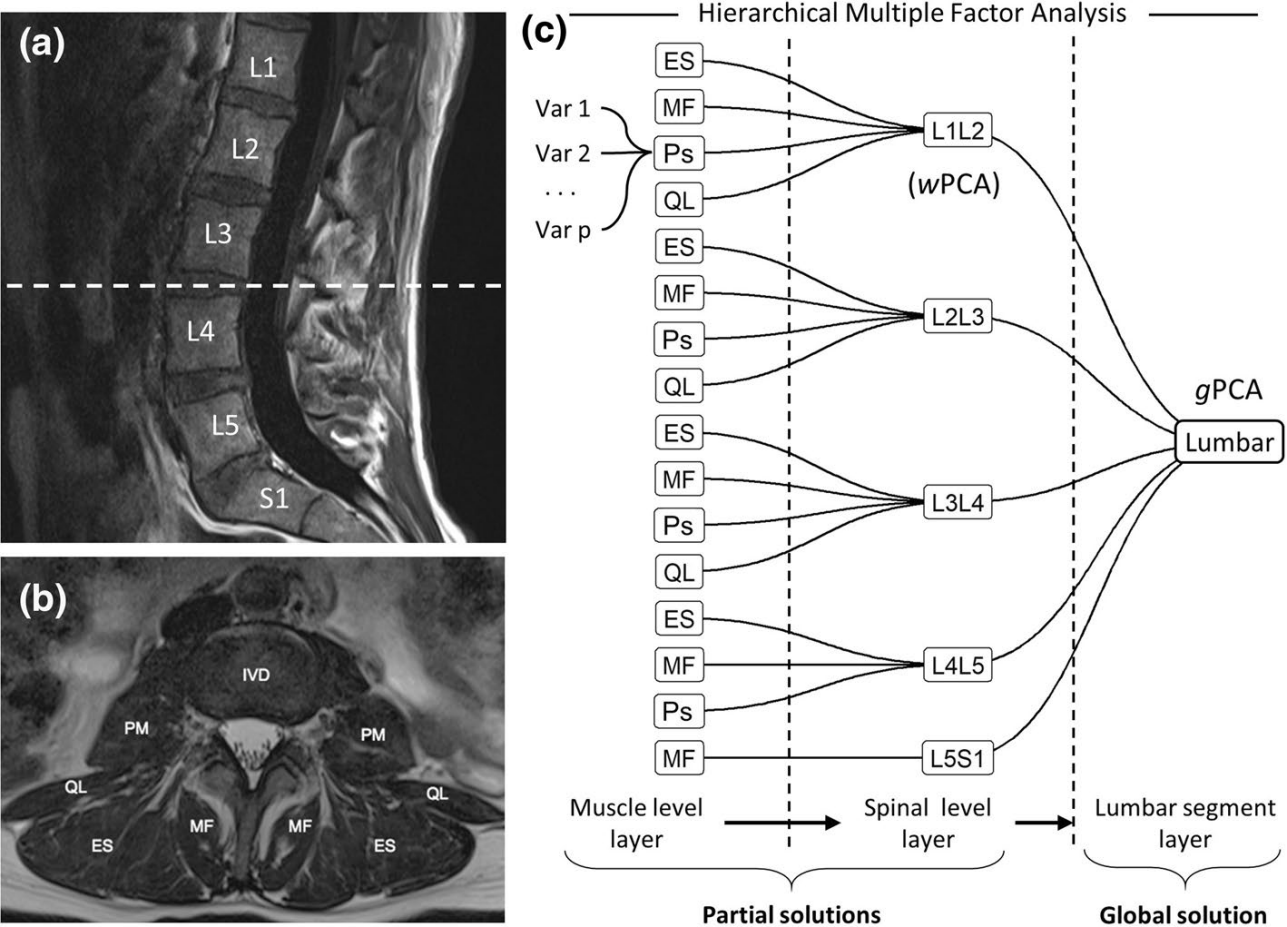
Hierarchical Multiple Factor Analysis for integrating spine MRI data. Sagittal (**a**) and cross-sectional (**b**) MRI planes of the lumbar spine with annotations of the vertebral bodies and paraspinal muscles. The data extracted from these MRI presented hierarchically, where variables are extracted for each paraspinal muscle from the cross-sectional image, muscles are nested into the specific spinal level, and the spinal levels are nested into the lumbar segment. Hierarchical MFA is an extension of PCA that weights the contribution of the variables in the analysis based on the tree nested structure defined (**c**). This allows for the extraction of patterns of variable associations (i.e., principal components) at the lumbar segment layer, at the spinal level layer and at the muscle layer. MF: multifidus, Ps: psoas, ES: erector spinae, QL: quadratus lumborum; PCA: principal component analysis, wPCA: weighted PCA, gPCA: global PCA, MFA: multiple factor analysis, IVD: intervertebral disk

Multivariate analytical approaches such as principal component analysis (PCA) and related methods have been previously used to integrate and reduce MRI data in an unsupervised learning context [8]. PCA extracts composited variables from all considered measures, known as principal components, which summarizes the relationship between measures, and determines how similar subjects are to each other considering all their values. Variables from lumbar PSM MRI data usually present with a hierarchical or nested relationship where several measures are obtained for each muscle, muscles are nested into the spinal level, and spinal levels are nested to the entire lumbar spinal segment (Fig. 1). This nesting of the variables constitutes a challenge for PCA since it ignores the intrinsic correlation of the hierarchy. With the goal to determine combinations of measures from PSM MRI data in the context of LBP, we used hierarchical multiple factor analysis (hMFA), an extension of PCA that takes into consideration the nested correlations in the data [9, 10]. Given that hMFA builds upon PCA methodologies, similar analytical tools and interpretations can be used in these methods, yet providing additional properties and metrics tailored to the challenges of spinal MRI data.

We analyzed PSM MRI data from two cohort datasets with distinct study designs [11, 12] to demonstrate the utility of hierarchical integrative unsupervised learning through hMFA on extracting clinically meaningful associations. We hypothesized that multivariate summaries of PSM MRI measures would associate with the presence of LBP symptoms (i.e., pain intensity). In this context, we used hMFA of PSM for three different use cases: (1) predicting longitudinal changes in pain using combinations of baseline PSM structure and quality, (2) integrating baseline and post-spaceflight MRI to assess longitudinal change in PSM structure and quality, and (3) integrating mixed-measures of PSM quality and spinal pathology between LBP patients and controls. Our findings show how hMFA can be used to distill large and complex dataset from spinal MRI into multivariate summaries that enhance the clinical utility of MRI-based biomarkers and provide summary metrics that motivate further exploration.

## Methods

### Datasets

We used data from two studies previously described [11, 12]. Dataset 1 contains features from lumbar MRI sequences. Localizer images, sagittal and axial T2-weighted images, and sagittal T1-weighted images were acquired for 12 NASA astronauts taken before spending 6-months in prolonged microgravity at the International Space Station and after returning to Earth [11, 13, 14]. For this current study, we analyzed multifidus (MF), erector spinae (ES), quadratus lumborum (QL), and psoas (Ps) bilaterally at each spinal segment between L1-S1. For each muscle measurement, we calculated total cross-sectional area (CSA) and percentage of functional cross-sectional area (FCSA) as the ratio lean muscle/CSA (representing a measure of muscle quality). Specifically, individual muscles were manually segmented and lean muscle was quantified by thresholding on T2-weighted MRIs (original segmentation and thresholding method by Fortin et al. [15] and described in [11, 13]. Functional cross-sectional area was calculated based on the fraction of lean muscle area to total cross-sectional area for a given muscle within an MRI slice. The average and absolute difference (asymmetry) between left and right muscle were calculated for CSA and FCSA. A total of 64 PSM MRI variables were considered for each timepoint (Table 1). Additionally, pain scores using the Visual Analog Scale (VAS) were obtained at baseline (pre-spaceflight) and post-spaceflight. Dataset 2 contains features from lumbar MRI sequences for 38 chronic LBP subjects and 14 asymptomatic controls [12]. Beyond standard clinical MRI, the protocol includes an advanced water-fat sequence for estimating fat fraction (Iterative Decomposition of water and fat with Echo Asymmetry and Least-Squares Estimation; IDEAL). For this current study, we analyzed MF and ES bilaterally at each disk segment between L1–S1. For each muscle measurement, we calculated mean fat fraction performed on the water-fat sequence (representing a measure of muscle quality; [10]). In addition, advanced sequences for cartilage endplate detection where performed (ultrashort echo time, UTE; [16]). The presence or absence of endplate pathology within either superior or inferior endplates and Modic change adjacent to either superior or inferior endplates at a given lumbar disk level were included in the analysis. This constitutes a mixed type dataset with both quantitative and qualitative variables (Table 2). Due to missing data in some of these metrics, we included 48 subjects.

**Table 1:** Dataset 1 PSM MRI variables included in the analysis for each one of the muscles and spinal segment combination: multifidus (MF; L1–S1), erector spinae (ES; L1–L5), psoas (Ps; L1–L5), and quadratus lumborum (QL; L1–L4). A total of 64 MRI variables are defined (4 for each combination of muscle and spinal segment)

Variable	Image source	Definition
%FCSA_asym	T2	Percentage of lean muscle calculated as the fraction of lean cross-sectional area respect to total area. Asymmetry between left and right muscle calculated as the absolute difference
CSA_asym	T2	Total cross-sectional muscle area. Asymmetry between left and right muscle calculated as the absolute difference
%FCSA_avg	T2	Percentage of lean muscle calculated as the fraction of lean cross-sectional area respect to total area. Average between left and right muscle
CSA_avg	T2	Total cross-sectional muscle area. Average between left and right muscle

**Table 2:** Dataset 2 MRI variables included in the analysis for each one of the spinal segments L1-S1. A total of 19 MRI variables are defined

Variable	Image source	Definition
multifidusFF	IDEAL	(Quantitative). Fat fraction of multifidus cross-sectional area. Average between left and right muscle
erectorspinaeFF	IDEAL	(Quantitative). Fat fraction of erector spinae cross-sectional area. Average between left and right muscle
CEPdx	UTE	(Qualitative). Whether there was presence (1) or absence (0) of Cartilage Endplate damage within either superior or inferior endplates at a given lumbar disk level
MC	T1 & T2	(Qualitative). Whether there was presence (1) or absence (0) of Modic Changes within either superior or inferior endplates at a given lumbar disk level

### Use cases

Use Case 1 on Dataset 1 investigates whether baseline PSM quality measured from MRI relates to changes in pain scores between baseline and post-spaceflight. Use Case 2 also uses Dataset 1, combining baseline and post-spaceflight PSM MRI data for determining global changes in PSM from baseline to post-spaceflight that might relate to changes in pain scores. Use Case 3 analyses Dataset 2 aiming at the questioning whether the presence of pathology (endplate defects and Modic changes) in adjacent tissues to PSM quality associates with pain.

### Hierarchical multiple factor analysis

MFA is an extension of PCA for multi-table analysis [17, 18]. In brief, MFA generate weights of each table as the first eigenvalue from a separate PCA of each table, which captures the major source of variance in each table. Each table is then normalized by dividing each entry by its weight (first eigenvalue) and solving a global PCA of concatenated normalized tables column-wise, which is equivalent to solving a generalized singular value decomposition using the weight vector as the column weights [17]. Extending to its hierarchical form (hMFA), the weights of one layer are a combination of the weights of its nested layers [9, 10, 17] (Fig. 1). The resulting eigenvectors, eigenvalues, loadings, and scores from a global weight-normalized PCA are the solution of the hMFA. A global solution is defined by taking in consideration all the variables into the analysis, while partial solutions of specific partitions of the hierarchy can also be obtained. In the analysis of Dataset 2 with mixed data types, hMFA was initiated from a nonlinear PCA for mixed data types (PRINCALS with monotonic b-splines transformation of degree 2, and 3 knots placed to the tertials of each fat fraction variable) for each spine level to determine optimal scaling and nonlinear transformations of quantitative and qualitative variables simultaneously [19]. The transformed tables were used as input for hMFA. In addition to the global solution loadings (correlation of each original variable to the extracted components), loadings corresponding to a specific table and hierarchical layer are equally interpretable. This allows for extracting partial set of loadings and scores (projection of the original data into the extracted component). For a mathematical description of the procedure see [17, 18, 20].

### Statistical analysis

All analysis has been coded in R 4.1.0 [21] on a Windows 10 machine. All data preparation was performed using different packages in the *tidyverse* meta-package [22]. hMFA was performed using *FactoMineR::HMFA()* [23]. Nonlinear PCA was fitted using *Gifi::princals()* [19]. To determine the association between different component scores and external variables to hMFA, linear regression (fixed and mixed effects) models were used (*lm()* function; LM or *lme4::lmer()* function; LMM). For statistical inference of each term in the models, either a Wald test or ANOVA F-test were used. MANOVA was performed using the *manova()* function. A p-value below 0.05 was considered statistically significant. All visualizations have been build using *ggplot2* [24] and *patchwork* [25] packages.

## Results

### Use case 1: predicting longitudinal changes in pain using combinations of baseline PSM structure and quality

**Fig. 2:**
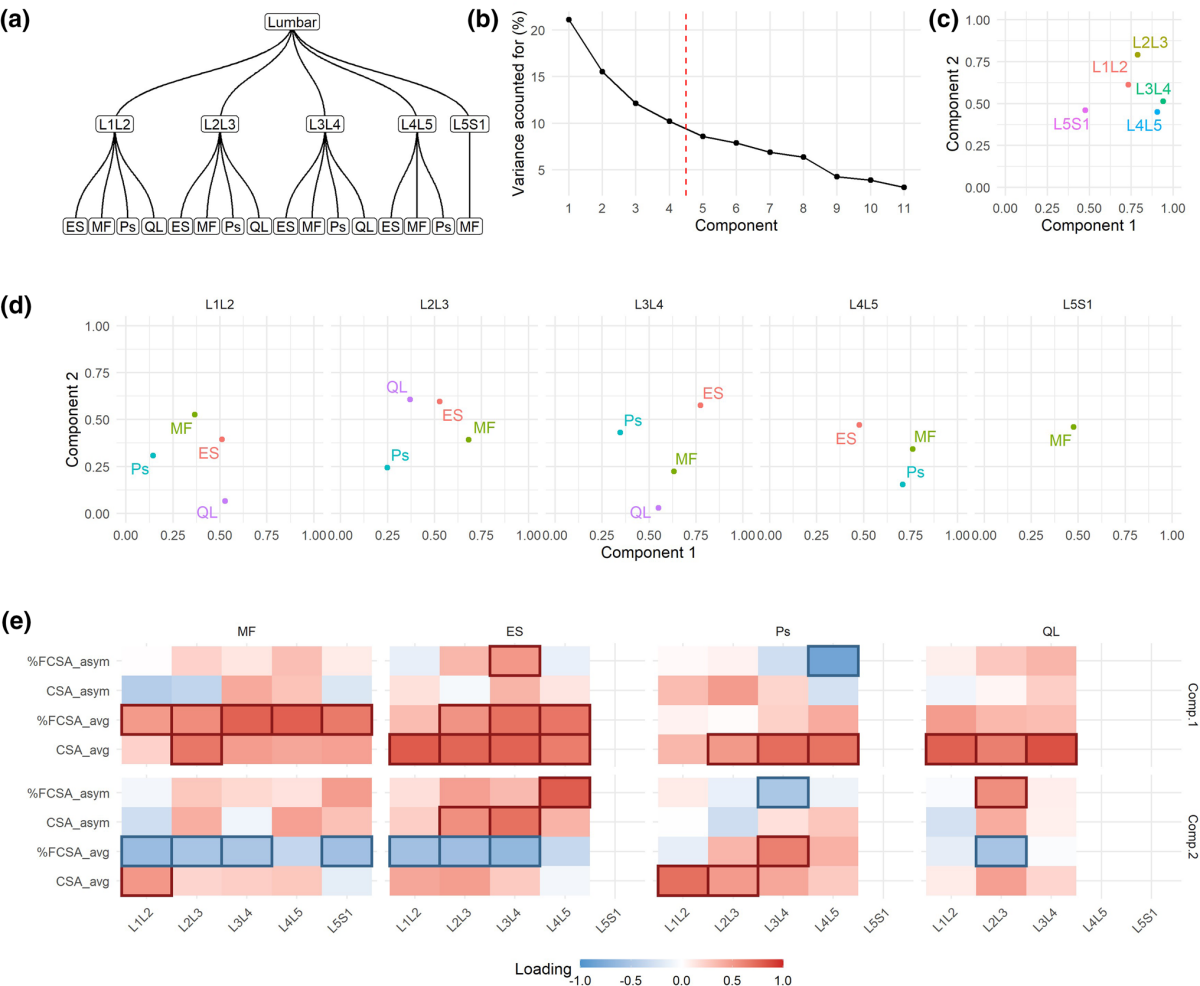
hMFA solution of Use Case 1. In Use Case 1, the hierarchical tree (**a**) was defined as in Fig. 1. From the global solution, we retained the first four components based on their percentage of variance accounted for (**b**). Comp. 1 explained 21.08%, Comp. 2 15.53%, Comp. 3 12.12%, and Comp. 4 10.22% of the total variance. The contribution of each layer into the definition of the components can be extracted, aiding with the interpretation of the results. For example, at the lumbar segment, the contribution of the variables conforming each spinal level show that the first component was most related to L3L4 and L4L5 variables, while L2L3 dominated in the second component (**c**). Equally, the contribution of the group of variables from the same muscle to the definition of the spinal level layer can be interpreted (**d**). In addition, the loadings (correlation between each variable and component) also aids interpretability by focusing on those loadings that has major absolute value (|loading|> 0.5) (**e**). MF: multifidus, Ps: psoas, ES: erector spinae, QL: quadratus lumborum

In Use Case 1, the defined hierarchical structure (Fig. 2a) is the same as illustrated in Fig. 1, that is a bottom layer relating variables for the same muscle, a middle layer of spine levels and an upper layer of lumbar segment. Thus, the components at the global solution defines the multi-segmental lumbar region (it considers all variables included in the analysis). Given the amount of variance explained by each component (Fig. 2b), the first four components were retained for further consideration. In using hMFA, we can calculate how much a partition of the hierarchy contributes to the variance explained by a component. Considering the spinal levels, we observed that L3L4 and L4L5 are the highest contributors to component 1, while L2L3 and L1L2 are the higher contributors to component 2 (Fig. 2c). L5S1 has the smallest overall contribution to the first two dimensions. Similarly, we can evaluate the contribution of each muscle by spine level combination (Fig. 2d). For instance, we find ES is the major contributor of the variance observed at L3L4 captured by components 1 and 2. The loadings, the correlation of each variable to a component, can also be interpreted at the global or partial solutions, aiding in explaining the components (Fig. 2e; Sup. Figure 1 and 2).

**Fig. 3:**
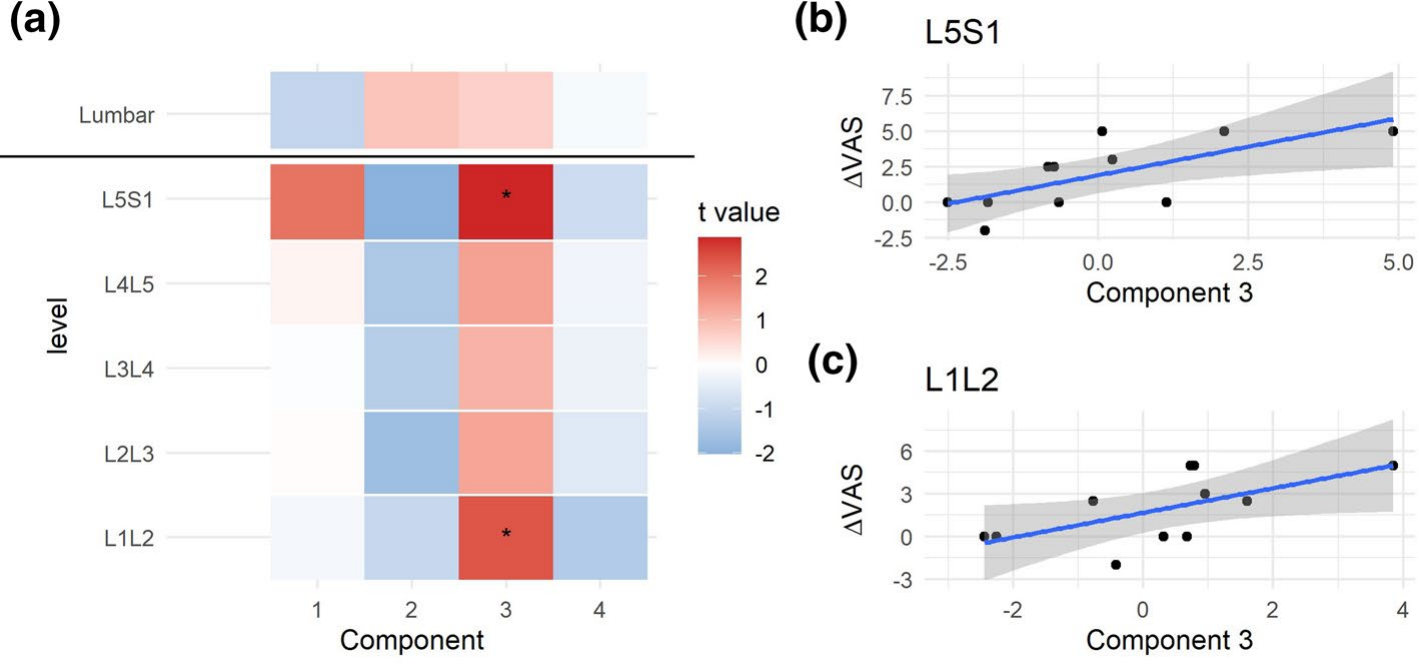
Integrated pre-spaceflight spinal MRI relates to changes in pain scores. For each of the partial components and the consensus components from the pre-spaceflight hMFA, a score representing the value that each subject gets for each one of the components can be obtained. These scores were used in linear models (LM) to predict changes in VAS (post- to pre-spaceflight; ΔVAS). The resulting t statistic for each LM is represented in (**a**) as a heatmap. At the lumbar segment layer (consensus hMFA components), none of the extracted components showed a significant association with ΔVAS. At the individual levels, the second component showed a negative association with ΔVAS, while the third components showed a positive association. These reached statistical significance at L5S1 (*p* = 0.018, **b**) and L1L2 (*p* = 0.041, **c**)

The coordinate of an individual into a component (component scores; Sup. Figure 3) can be extracted at the global and partial solutions, summarizing in single-number metrics the degree at which each individual relates to the associations of variables captured by the loadings. We examined whether the different hMFA components at baseline could predict changes in pain scores using linear models (ΔVAS from baseline to post-spaceflight; Fig. 3). At the global solution, none of the retained components showed significance at predicting ΔVAS. At the spinal level, the second component, although not significant, showed moderate negative associations with ΔVAS at the levels between L2L3 to L5S1. At the third component, higher scores correlate with higher ΔVAS, being significant at the L1L2 and L5S1 spinal segments (Fig. 3b and c). The loadings at L1L2 for component 3 (Sup. Figure 2) relate subjects with higher asymmetry in percent muscle composition at MF with lower asymmetry in Psoas area, while L5S1 loadings are mainly capturing high values in MF cross-sectional area. This suggest that subjects with high L1L2 asymmetry between left and right MF lean muscle composition and with little differences between left and right Psoas area, and/or higher average MF CSA at L5S1 are more likely to increase in pain reported outcome after prolonged time spent in microgravity.

### Use case 2: temporal integration of baseline and post-spaceflight MRI to assess longitudinal change in PSM structure and composition

**Fig. 4:**
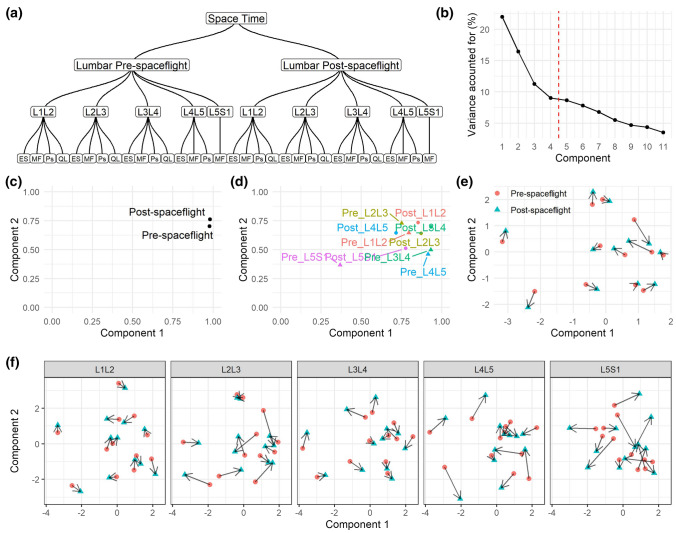
hMFA solution Use Case 2. In Use Case 2, the hierarchical tree (**a**) was defined similarly to that in Use Case 1 (Fig. 2a) but with the addition of the post-spaceflight data and a temporal layer (time in space). From the global solution (space time), we retained the first four components based on their percentage of variance accounted for (**b**). Comp. 1 explained 21.97%, Comp. 2 16.42%, Comp. 3 11.25%, and Comp. 4 9.02% of the total variance. The contribution of each layer into the definition of the components can be extracted (**c–d**). With the incorporation of the temporal layer, it is possible to determine the changes in the component scores for each subject between pre- and post-spaceflight. These can be calculated at the lumbar segment level (**e**) and for each individual spinal level (**f**). Each arrow indicates the change that each subject had in the respective component from pre to post-spaceflight. The magnitude of the change can be calculated and used for further analysis. MF: multifidus, Ps: psoas, ES: erector spinae, QL: quadratus lumborum

Here we used the same dataset as Use Case 1, but we added an extra layer to incorporate a longitudinal component. The principal goal was to compare changes between baseline and post-spaceflight in the hierarchical structure of the PSM MRI variables (Fig. 4a). The first four components were retained for analysis (Fig. 4b). The influence of baseline and post-spaceflight tables is balanced in component 1 and 2 (Fig. 4c). When considering the variables at the individual spine level, L5S1 baseline contributed far less to the definition of components 1 and 2 than the other levels (Fig. 4d). We calculated the amount of change in the component scores pre- and post-spaceflight (Fig. 4e and f), observing that the major change between baseline and post-spaceflight occurs at L5S1.

**Fig. 5:**
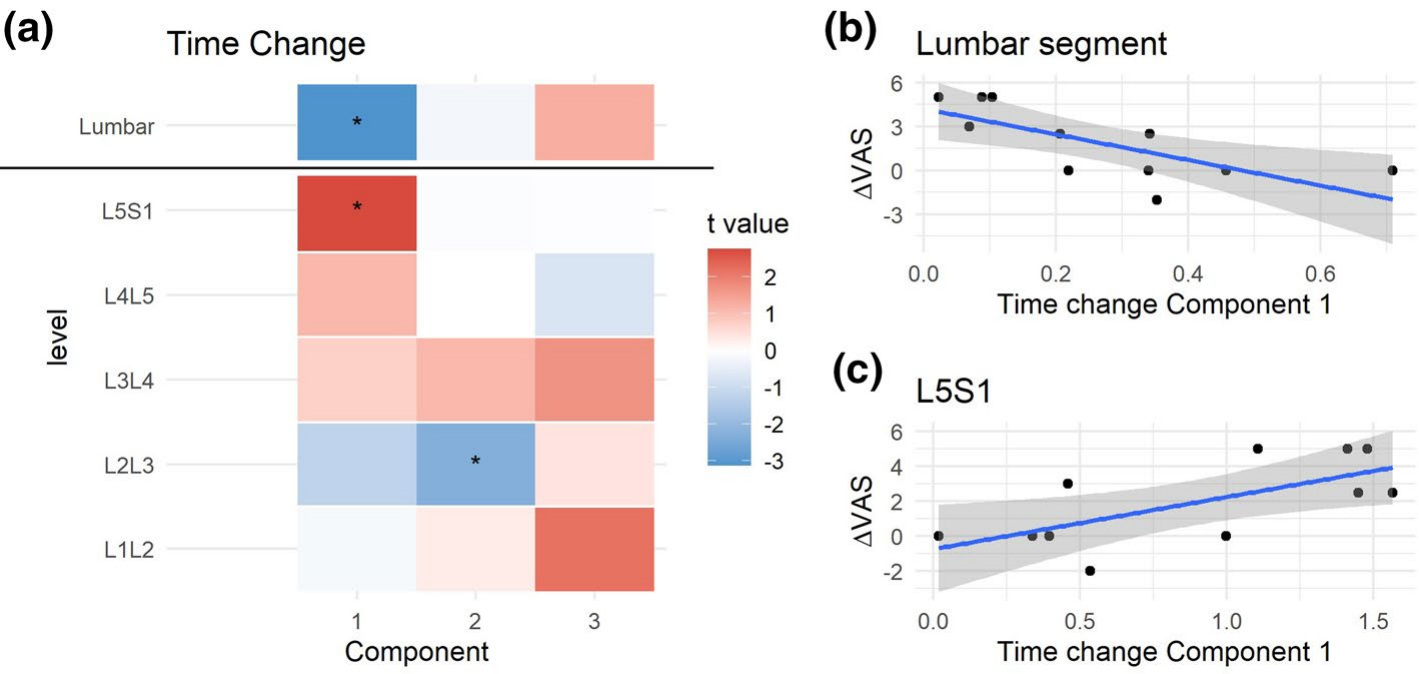
Integrated pre to post-spaceflight spinal MRI changes relates to changes in pain scores. For each of the partial components and the consensus components from the Use Case 2 hMFA, the subject scores were used in linear models (LM) predicting changes in VAS (post- to pre-spaceflight; ΔVAS). The resulting t statistic for each LM is represented in (**a**) as a heatmap. At the lumbar segment layer (consensus hMFA components), component 1 showed a significant association with ΔVAS (*p* = 0.011, **b**). At the individual levels, component 1 showed a positive association with ΔVAS at L5S1 (*p* = 0.022, **c**), while component 2 showed a significant negative association at L2L3 (*p* = 0.047)

We investigated whether the changes in component scores over time related to changes in ΔVAS (Fig. 5). We observed that subjects with little change in component 1 were the patients with major ΔVAS (Fig. 5b). This relationship is inverted when considering scores at the L5S1 level only (Fig. 5c). The loadings of component 1 at the lumbar segment level (Sup. Figure 4) indicate global changes in PSM asymmetry across levels from baseline to post-spaceflight, while L5S1 loadings indicate increase in muscle cross-sectional area and quality of the MF after spaceflight.

### Use case 3: integrating mixed measures of PSM quality and spinal pathology between patients and controls

**Fig. 6:**
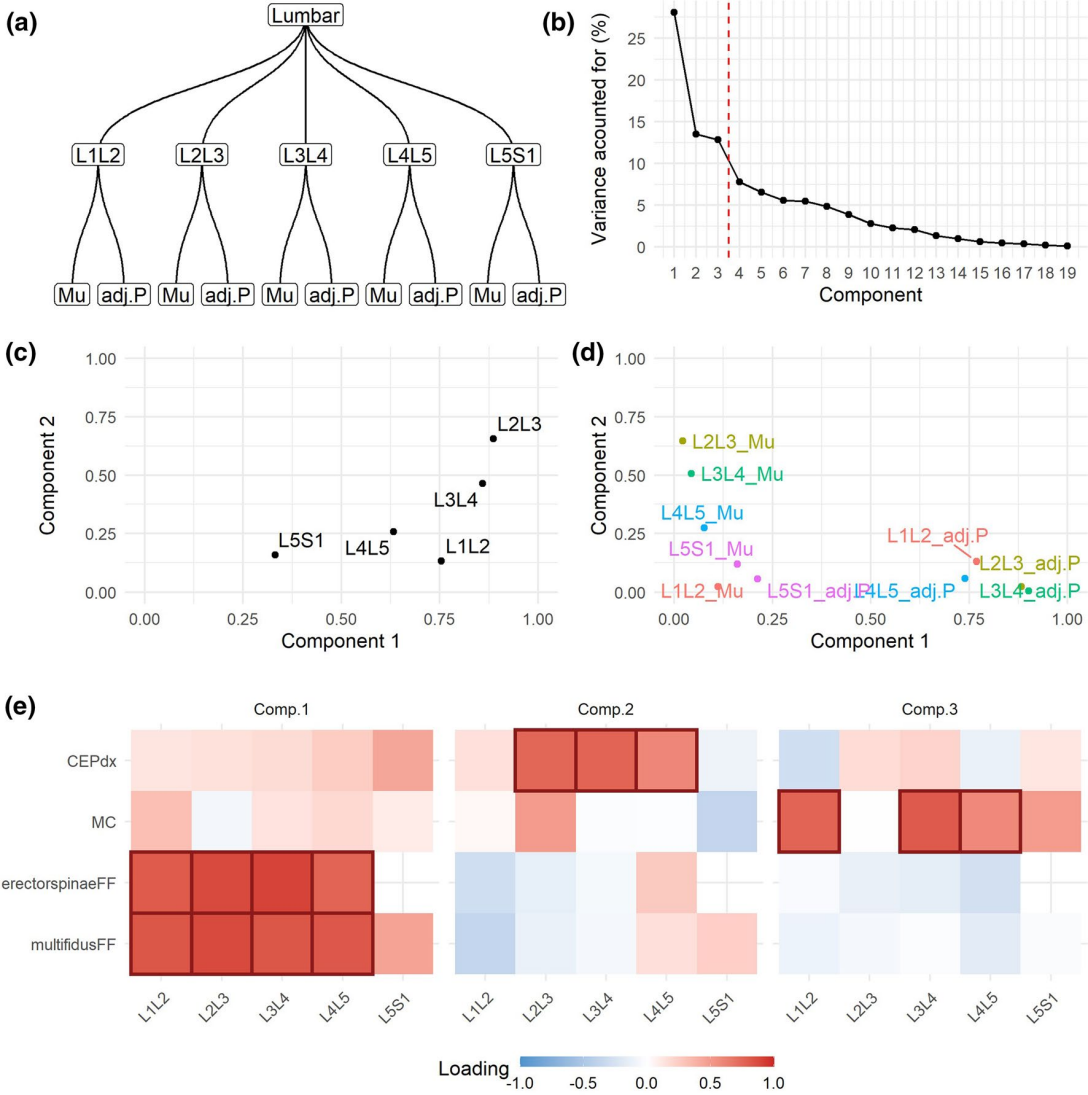
hMFA solution for Use Case 3. The Dataset 2 for Use Case 3 consists of a mix of quantitative (muscle fat fraction, Mu) and Qualitative (adjacent pathology, adj.P) variables. For the analysis, they were considered as its own layer (variable origin level) nested into the spinal level and the lumbar segment (**a**). A NL-PCA was used to transform and quantify the variables at each level (linearization) as pre-processing to hMFA. We retained the first three components based on their percentage of variance accounted for (**b**). Comp. 1 explained 28.62%, Comp. 2 21.78%, and Comp. 3 15.94% of the total variance. The contribution at the spinal level (**c**) and at the variable origin level (**d**), and the loadings (**e**) are shown. |loadings|> 0.5 are marked

In Use Case 3 we co-analyzed PSM quality (in this case, fat fraction) along with the presence of adjacent tissue pathology (endplate pathology and Modic changes) in a cohort of chronic LBP patients and asymptomatic controls. Variables specify a lower layer of variable origin (PSM quality vs. pathology), a middle layer of spine levels and an upper layer of lumbar segment (Fig. 6a). The resulting first three dimensions were retained for further analysis (Fig. 6b). Variables at L2L3 had the major contribution into the definition of the first two dimensions, followed by L3L4, while variables in L5S1 contributed the least (Fig. 6c). At the variable origin level, there is apparent independence between adjacent pathology presence (contributing to component 2 and 3) and PSM quality (contributing to component 1). This is further evidenced by the loadings (Fig. 6e), indicating that there is little relationship between the presence of pathology and PSM quality in the data.

**Fig. 7:**
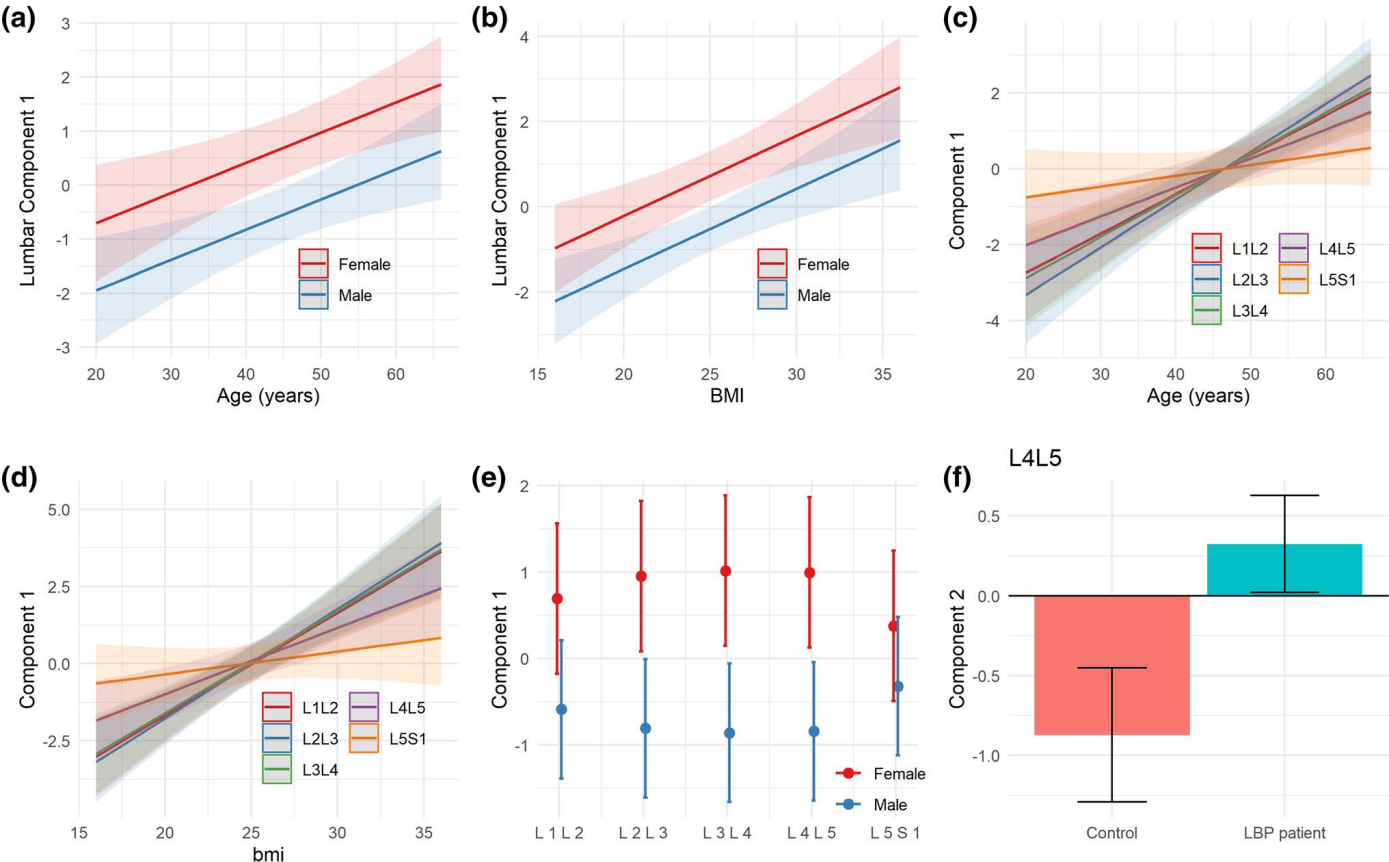
Explaining hMFA Use Case 3 component 1. The component 1 at the lumbar segment in hMFA Use Case 3 related to muscle fat fraction was significantly associated with age, BMI and sex (LM: age *p* = 0.003, BMI *p* < 0.001, sex *p* = 0.003; **a–b**). Age was significantly associated with component 1 at all spinal levels (**c**), although a significant interaction was observed, indicative that the magnitude of the relationship (slope) was different across spinal levels (LM: Age *p* < 0.001, Level *p* < 0.001, Age *x* Level *p* < 0.001). Very similar results were found for BMI (**e**, LM: Age *p* < 0.001, Level *p* < 0.001, Age *x* Level *p* < 0.001). In the case of sex (**d**) a main effect of sex was significant, but there were no significant differences across levels nor interaction between sex and levels (LM: Sex *p* = 0.0049, Level *p* = 0.99, Age *x* Level *p* = 0.11). Component 2 at L4L5 was significantly different between controls and LBP patients (**f**, LM: *p* = 0.038)

No statistical differences were found between patient and controls considering the first three components at the global solution together (MANOVA *p* = 0.26), suggesting that the variance induced by the presence of pathology and PSM quality across the lumbar segment cannot be explained by the presence or absence of pain. Component 1 explaining muscle quality was correlated to age, body mass index (BMI) and sex at the lumbar segment layer, as well as at each individual spine level (Fig. 7), while the other three components did not show such association. Component 2 capturing CEP damage was significantly different between LBP patients and controls at L4L5. No other component at the global nor partial level was found to distinguish between patients and controls.

## Discussion

We organized PSM MRI data from two separate datasets with different study designs based on the nested multi-level and multi-muscle relationships of the lumbar spine (Fig. 1). A major advantage of using hMFA is the ability to compute a global solution, which considers the association of all variables in the analysis by balancing their contribution according to the defined hierarchy. In addition, partial solutions at different partitions of the hierarchy (e.g., for each spinal level) can be obtained, providing summaries at different levels of the lumbar spine, aiding in the analysis and interpretation of the results (Fig. 1). We hypothesized that multivariate summaries of PSM MRI measures would associate with the presence of LBP symptoms. From three distinct Use Cases study designs, we demonstrate the application of hMFA for the integration and study of multi-level and multi-muscle MRI data in an unsupervised learning framework in relation to LBP. We show that hMFA can be used considering the lumbar spine a nested multi-segmental unit as opposed to a series of discrete and isolated spine segments, leveraging the intra- and inter-level anatomical associations, and confirmed our primary hypothesis that integrated summaries from PSM MRI associate and predict pain.

Prior multivariate analytical approaches used for MRI data include principal component analysis (PCA) [8]. However, variables from spinal MRI data usually present with a hierarchical or nested structure which constitutes a challenge PCA and therefore the need for multi-table approaches. MFA has been previously used to combine features extracted from different MRI sequences for diseases like multiple sclerosis [26]. Additionally, MFA has been used for integrating MRI data with other data types such as patient symptoms into a singular analysis [27]. There is little work published on the use of multivariate methods for integrative spinal MRI data, and to our knowledge we are the first to demonstrate the utility of a hierarchical multi-table method. Khan et al., applied PCA and factor analysis (not to be confused with factorial methods such MFA) to lumbar spinal MRI to uncover multidimensional patterns associated to aging [28]. Using several lumbar spinal features measured at different vertebral levels, they obtained a first principal component that correlated with participant age, similar to what we observed in Use Case 3. The use of hMFA allows for more than the single solution provided by PCA. The capacity to study components and variables at a different level of the hierarchy provides insights to the anatomical relations in the multi-segment structure of the spine.

The value of hMFA is clearly illustrated in our three use cases. In Use Case 1, the hMFA solution at the lumbar level did not show predictive association with ΔVAS, whereas different degrees of association could be found at the spinal levels, suggesting that PSM structure and quality before prolonged microgravity can predict the development of pain. In Use Case 2, we were able to study the temporal changes at the global lumbar as well as the individual spinal levels. Although further analysis would be needed, the changes observed in relationship with increase in pain scores illustrates how hMFA can guide data interpretation and hypothesis generation in complex spinal MRI datasets. For example, changes between baseline to post-spaceflight MF quality at L5S1 relate to increase in pain after prolonged microgravity. Interestingly, some subjects change in reducing MF quality, while some individuals increase it after spaceflight, but it is the magnitude of change that relates to pain. One could hypothesize that any change in muscle quality promoted by prolonged microgravity could disrupt anatomical structures and in turn, spinal stability, which could cause impairment and pain [11, 13]. Further, in Use Case 3, we demonstrated the incorporation of both quantitative and categorical variables into the analytical workflow. The analysis recapitulates previously known associations from this dataset [12] on the association of CEP damage and LBP diagnostic in an unsupervised fashion and considering the full data. It is worth noting our results are limited by the small sample size of the studies and the purpose of this paper is to demonstrate a novel approach for analyzing multiple muscle and multiple spinal-level PSM data from spinal MRIs. Further research in the relation of PSM with pain is required.

Integrative data reduction methodologies associated with hMFA can leverage all available information from spinal MRI. These techniques distill complex datasets into summary statistics representing associations of variables and groups of variables at different layers of the anatomical structure, and at the level of the individual in the multidimensional space. These metrics can be used for further analytical goals such as predictive modeling, determining patient phenotypes through clustering, or building multivariable MRI-based biomarkers, as well as exploratory data analysis and hypothesis generation. Further research should be oriented to further develop the application of hMFA and other multi-table methods on determining the complex pathophysiology of LBP from integrated spine MRI metrics.

## Supplementary Information

Below is the link to the electronic supplementary material.

Supplementary file1 (DOCX 2095 KB)

## Abbreviations

CEP: Cartilage endplate
CSA: Cross-sectional area
ES: Erector spinae
FCSA: Functional cross-sectional area
hMFA: Hierarchical multiple factor analysis
LBP: Low back pain
MC: Modic change
MFA: Multiple factor analysis
MFA: Multifidus
MRI: Magnetic resonance imaging
PCA: Principal component analysis
PSM: Paraspinal muscle
QL: Quadratus lumborum
VAS: Visual analog scale

## References

[1] Sheehan NJ (2010). Magnetic resonance imaging for low back pain: indications and limitations. Ann Rheum Dis.

[2] Wassenaar M, van Rijn RM, van Tulder MW (2012). Magnetic resonance imaging for diagnosing lumbar spinal pathology in adult patients with low back pain or sciatica: a diagnostic systematic review. Eur Spine J.

[3] Roudsari B, Jarvik JG (2010). Lumbar spine MRI for low back pain: indications and yield. AJR Am J Roentgenol.

[4] Chou D, Samartzis D, Bellabarba C (2011). Degenerative magnetic resonance imaging changes in patients with chronic low back pain: a systematic review. Spine.

[5] Hodges PW, Bailey JF, Fortin M, Battié MC (2021). Paraspinal muscle imaging measurements for common spinal disorders: review and consensus-based recommendations from the ISSLS degenerative spinal phenotypes group. Eur Spine J.

[6] Maher C, Underwood M, Buchbinder R (2017). Non-specific low back pain. Lancet.

[7] Koes BW, van Tulder MW, Thomas S (2006). Diagnosis and treatment of low back pain. BMJ.

[8] Haefeli J, Mabray MC, Whetstone WD (2017). Multivariate analysis of MRI biomarkers for predicting neurologic impairment in cervical spinal cord injury. AJNR Am J Neuroradiol.

[9] Le Dien S, Pagès J (2003). Hierarchical multiple factor analysis: application to the comparison of sensory profiles. Food Qual Prefer.

[10] Pagès J, Pagès J (2014). Hierarchical multiple factor analysis. Multiple factor analysis by example using R.

[11] Bailey JF, Nyayapati P, Johnson GTA (2021). Biomechanical changes in the lumbar spine following spaceflight and factors associated with postspaceflight disc herniation. Spine J.

[12] Bailey JF, Fields AJ, Ballatori A (2019). The relationship between endplate pathology and patient-reported symptoms for chronic low back pain depends on lumbar paraspinal muscle quality. Spine.

[13] Bailey JF, Miller SL, Khieu K (2018). From the international space station to the clinic: how prolonged unloading may disrupt lumbar spine stability. Spine J.

[14] Chang DG, Healey RM, Snyder AJ (2016). Lumbar spine paraspinal muscle and intervertebral disc height changes in astronauts after long-duration spaceflight on the international space station. Spine.

[15] Fortin M, Battié MC (2012). Quantitative paraspinal muscle measurements: inter-software reliability and agreement using OsiriX and ImageJ. Phys Ther.

[16] Fields AJ, Han M, Krug R, Lotz JC (2015). Cartilaginous end plates: quantitative MR imaging with very short echo times—orientation dependence and correlation with biochemical composition. Radiology.

[17] Abdi H, Williams LJ, Valentin D (2013). Multiple factor analysis: principal component analysis for multitable and multiblock data sets: multiple factor analysis. WIREs Comp Stat.

[18] Escofier B, Pagès J (1994). Multiple factor analysis (AFMULT package). Comput Stat Data Anal.

[19] Mair P, Leeuw J, Groenen PJF (2019) Gifi: multivariate analysis with optimal scaling. http://r-forge.r-project.org/projects/psychor/

[20] Pages J, Escofier B, Haury J, Devillers J, Karcher W (1991). Multiple Factor analysis : a method to analyse several groups of variables measured on the same set of individuals. Applied multivariate analysis in SAR and environmental studies.

[21] R Core Team (2021) R: A language and environment for statistical computing. R foundation for statistical computing, Vienna, URL https://www.R-project.org/.

[22] Wickham H, Averick M, Bryan J (2019). Welcome to the tidyverse. Open Source Softw.

[23] Lê S, Josse J, Husson F (2008). FactoMineR: an R package for multivariate analysis. J Stat Softw.

[24] Wickham H (2016). ggplot2: elegant graphics for data analysis.

[25] Pedersen TL (2020). Patchwork: the composer of plots. R Package Version.

[26] Rebbah S, Delahaye D, Puechmorel S, et al (2018) A combined MRI biomarker approach using a non-standard multiple factor analysis. In: 2018 11th international congress on image and signal processing, BioMedical Engineering and Informatics (CISP-BMEI), pp 1–6

[27] Vilor-Tejedor N, Alemany S, Cáceres A (2018). Sparse multiple factor analysis to integrate genetic data, neuroimaging features, and attention-deficit/hyperactivity disorder domains. Int J Methods Psychiatr Res.

[28] Khan AA, Iliescu DD, Sneath RJ (2015). Principal component and factor analysis to study variations in the aging lumbar spine. IEEE J Biomed Health Inf.

