# Supplementary material for: Using hierarchical unsupervised learning to integrate and reduce multi-level and multi-paraspinal muscle MRI data in relation to low back pain
Source: Eur Spine J. 2022 Mar 25;31(8):2046–56. doi: 10.1007/s00586-022-07169-z (PMC9338899; doi:10.1007/s00586-022-07169-z)
Supplement: Supplementary file 1 — Supplementary file1 (DOCX 2095 KB) [file 586_2022_7169_MOESM1_ESM.docx]

**Supplementary Figures**


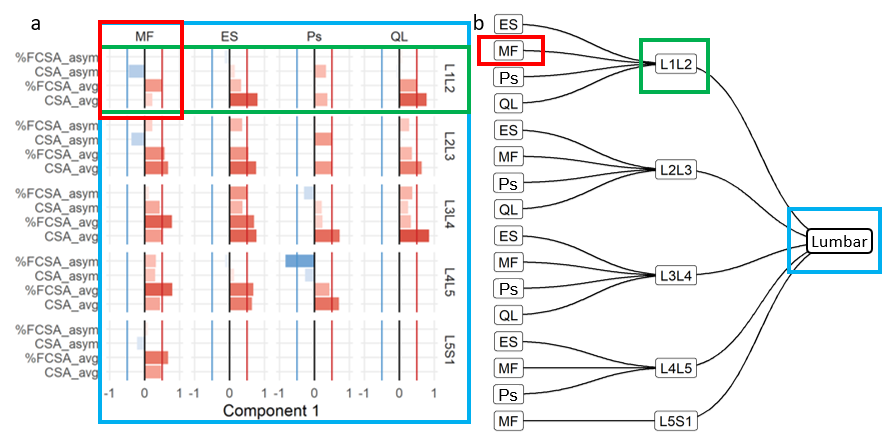


**Sup. Figure 1. Interpretation of loadings at each partition of analysis.** Using component 1 as an example, loadings (**a**) can be interpreted at the global solution (blue square) capturing the integrated relationship of all variables included in the analysis. The interpretation can also be partitioned at different levels of the hierarchy (**b**). For example, loadings of all variables included in L1L2 (green square; 4 variables per muscle, 16 variables total) can be interpreted as a unit to understand component 1 at this specific segment of the spine. Similarly, loadings at a single muscle for a single spinal level can be interpreted. For instance, we can consider the 4 variables of the multifidus at the L1L2 spinal level (red square) to understand the role of these variables in component 1 in the context of the muscle, then on the spinal level and finally on the lumbar spine.

**
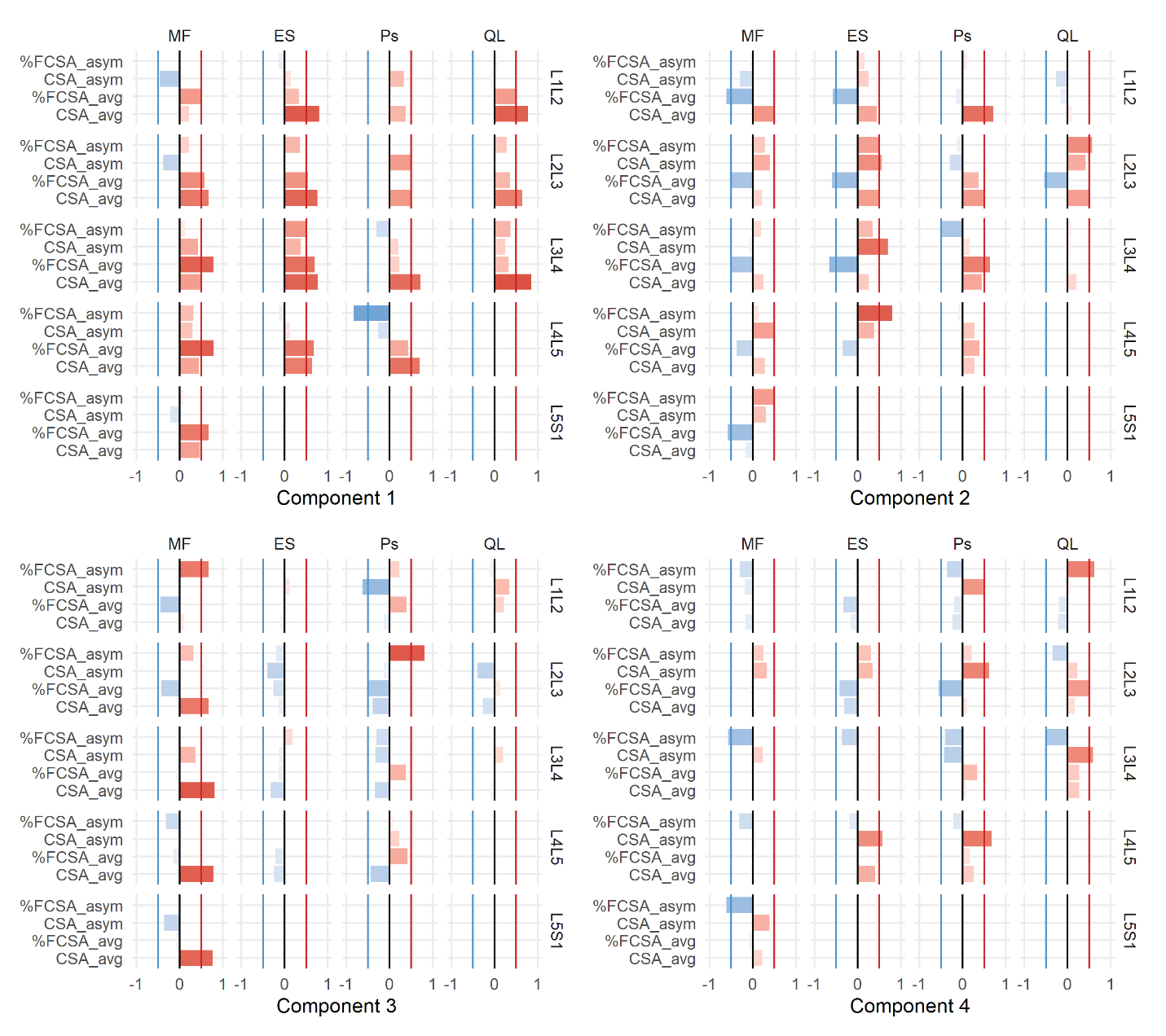
Sup. Figure 2. Use Case 1 variable loadings.** Each one of these panels represents the variable loadings for the first 4 components of the hMFA solution in Use Case 1. Blue (negative loading) and red (positive loading) vertical lines represents |loading| = 0.5.


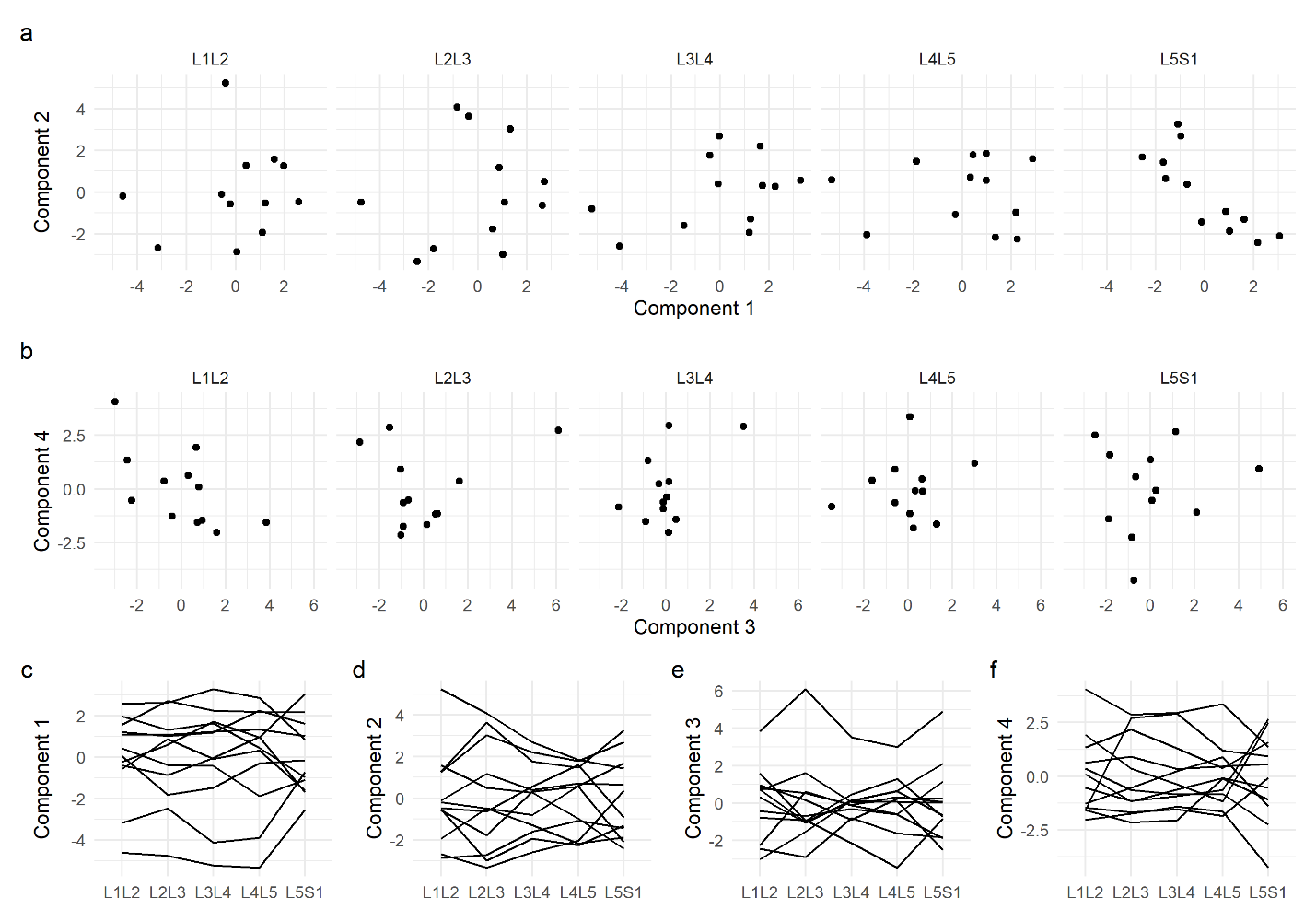


**Sup. Figure 3. hMFA subject scores for Use Case 1.** This figure shows the projection of the original data into the different spinal level partial solutions (partial subject scores). For each spinal level, the first component plane (component 1 and 2) is shown in (**a**) and the second component plane in (**b**). The values along the levels for each one of the components are shown in (**c-f**) where each line represents a subject and its variation along the spinal levels. This information can be used to determine “trajectories” of subjects with similar variations over the lumbar spine.


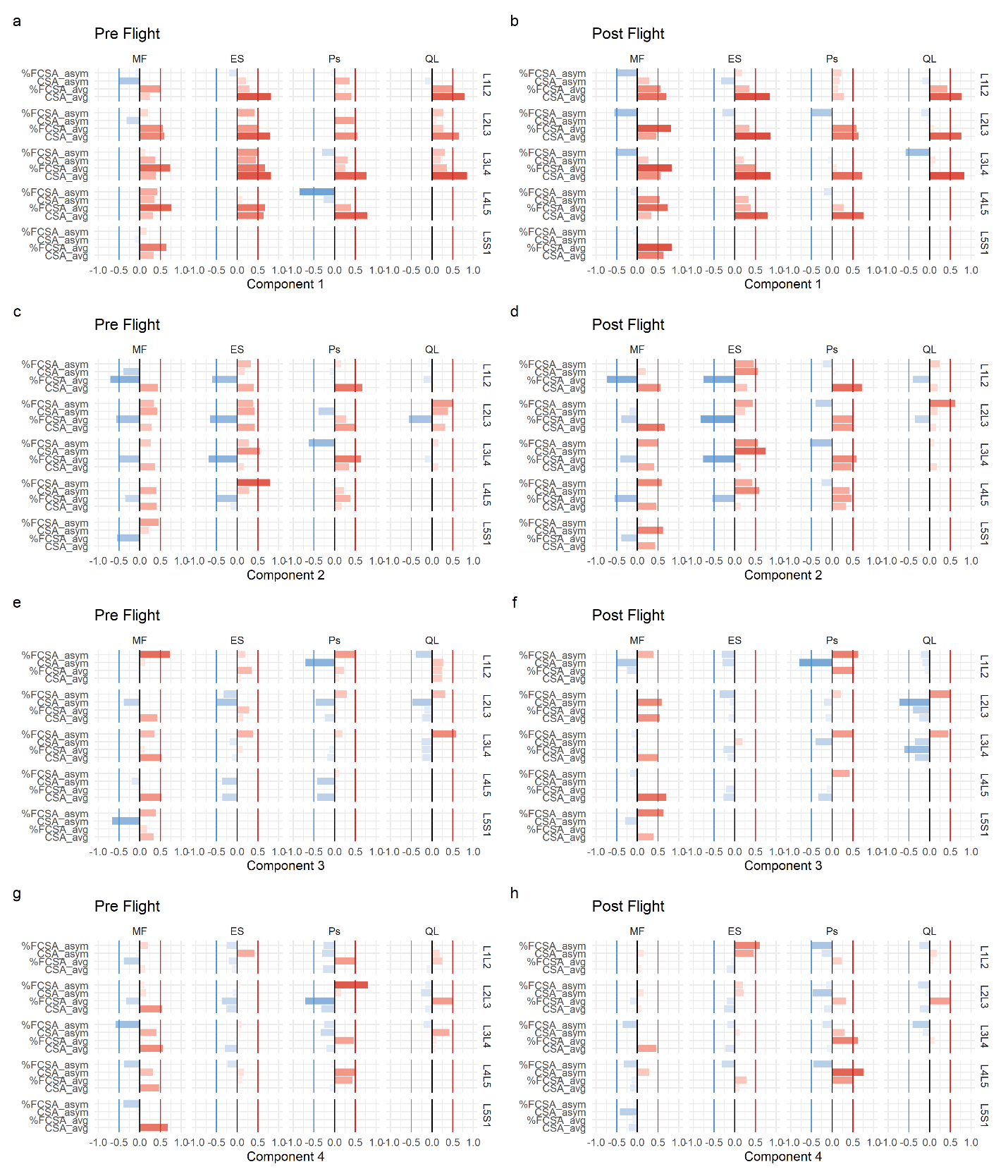


**Sup. Figure 4. Use Case 2 hMFA variable loadings.** For Use Case 2, loadings can be interpreted as in Use Case 1, with the addition of the time in space layer. Comparing pre to post-spaceflight loadings allows to interpret the changes in variable associations that happens after time in space. Blue (negative loading) and red (positive loading) vertical lines represents |loading| = 0.5.
